# Supplementary material for: Complexome profiling of the Chlamydomonas psb28 mutant reveals TEF5 as an early PSII assembly factor
Source: Plant Cell. 2025 Mar 17;37(6):koaf055. doi: 10.1093/plcell/koaf055 (PMC12164590; doi:10.1093/plcell/koaf055)
Supplement: koaf055_Supplementary_Data [file koaf055_supplementary_data.zip › psb28_interactive.zip!/plots/intensity/qualityPlots/peptides/_Cre11.g477950.t1.1.html]

### Cre11.g477950.t1.1

Trivial name:   
SeqCoverage [median]: 67.60  
Mass: 94.34 kDa  
  
 

| Sequence | MissedCleavage | Mass [Da] | wtIntensity | muIntensity | Score |
| --- | --- | --- | --- | --- | --- |
| AAAAASLGQAQDAAAQAQNTAAAAVGNAQTAAGDAAGK | 0 | 3323.61 | 978.44 | 1430.46 | 37.36 |
| AAAAQATAAAAGAQEAASAAAGQATATATDAMTK | 0 | 2961.41 | 753.65 | 13289.17 | 69.55 |
| AAAAQEAATAAAGQAAAVANNAATAASK | 0 | 2412.20 | 607.49 | 9108.64 | 98.93 |
| AAAASAQGALAAATGQAR | 0 | 1555.81 | 4369.55 | 15238.21 | 160.84 |
| AAADGAVANAQGAVTSFATK | 0 | 1819.91 | 0.00 | 10003.75 | 116.73 |
| AAAQAQEAAAGAATQAK | 0 | 1527.76 | 10960.52 | 15734.68 | 112.34 |
| AAEAQAAAAGAAEQAQAAAANAQQAAADAAGK | 0 | 2808.34 | 588.58 | 0.00 | 98.88 |
| AAEAQKAAEGAAAAAQAK | 1 | 1626.83 | 0.00 | 1345.85 | 77.51 |
| AAEGAAAAAQAK | 0 | 1028.53 | 8544.58 | 9962.18 | 228.20 |
| AAEGAVTAAQAK | 0 | 1086.57 | 7186.52 | 10489.77 | 131.44 |
| AAESAAASAAQAQATAASAVSAVQDK | 0 | 2345.15 | 8972.11 | 29236.36 | 166.64 |
| AAEVLAEVLAK | 0 | 1112.64 | 6204.87 | 17047.38 | 118.71 |
| AAEVLAEVLAKK | 1 | 1240.74 | 0.00 | 1940.97 | 57.02 |
| AALLVAIQAK | 0 | 996.63 | 748.17 | 6208.76 | 142.64 |
| AASAAAEQAQAAAVGAQQAAVEK | 0 | 2111.06 | 10833.58 | 23686.82 | 201.12 |
| AATVAADAQAAAAK | 0 | 1228.64 | 8465.52 | 14304.72 | 156.58 |
| ADDVAVQVGATAAGAAGAKPPAGVPK | 1 | 2288.21 | 397.00 | 5756.17 | 107.21 |
| AEDTAAAAQAQAAAAAESAR | 0 | 1843.87 | 2395.22 | 7521.80 | 137.73 |
| AGEVQAAAAGAAEQAQAQASAAAAEAQK | 0 | 2539.23 | 3369.87 | 17475.76 | 90.90 |
| AQDAAAGAAAAATEK | 0 | 1315.64 | 2637.61 | 4782.78 | 147.73 |
| AQEAAEGAAAAAQAK | 0 | 1356.66 | 5682.65 | 9329.40 | 198.14 |
| AQEAATGAAAK | 0 | 987.50 | 1907.77 | 1185.42 | 72.93 |
| AQETAAAAAGQAQAAAADAAAK | 0 | 1926.94 | 2885.54 | 8114.11 | 99.37 |
| AQGAATAATEQAK | 0 | 1216.60 | 2217.15 | 3389.67 | 126.12 |
| AQGAATAATEQAKDAAAAAQQQAAAAADAAK | 1 | 2811.37 | 0.00 | 1374.18 | 64.54 |
| ASEAATGALSEIAAVDGEGGAAK | 0 | 2044.99 | 0.00 | 1549.53 | 148.57 |
| DAAAAAQQQAAAAADAAK | 0 | 1612.78 | 813.82 | 2775.78 | 126.56 |
| DAAAGAAAQAQTAAAGLMGSLNAAAGK | 0 | 2328.15 | 0.00 | 3029.51 | 59.05 |
| GAAASAVGQATAQATAAVDQAK | 0 | 1956.99 | 5358.30 | 50388.33 | 175.65 |
| GQAEAAAAQAQNTAAAAVGNVQTAAADAAAK | 0 | 2781.36 | 1815.30 | 1335.03 | 80.93 |
| QAQETATQAEEAAK | 0 | 1474.69 | 4162.59 | 5376.89 | 179.19 |
| SLMYLDGPDVK | 0 | 1236.61 | 1107.35 | 6071.69 | 96.33 |
| TADYGTLEAAVDK | 0 | 1352.65 | 0.00 | 1761.50 | 97.46 |
| TADYGTLEAAVDKLVAAR | 1 | 1862.97 | 1493.62 | 0.00 | 42.65 |
| TGVEAAVR | 0 | 801.43 | 3254.56 | 8908.49 | 143.61 |
| VITSSDGVEAAIR | 0 | 1316.69 | 7317.81 | 24292.08 | 157.38 |
| VPAVVALLGTGASAAADPAAAEALR | 0 | 2261.24 | 1165.81 | 0.00 | 52.06 |
| YLAAHYAAEDPPLVYINHSAK | 0 | 2342.17 | 0.00 | 1311.15 | 37.55 |
|  |  |  | 117195.5 | 344757.0 |  |
